# The Changes in Color and Image Parameters and Sensory Attributes of Freeze-Dried Clones and a Cultivar of Red-Fleshed Apples

**DOI:** 10.3390/foods13233784

**Published:** 2024-11-25

**Authors:** Ewa Ropelewska, Mariusz Lewandowski

**Affiliations:** 1 Fruit and Vegetable Storage and Processing Department, The National Institute of Horticultural Research, Konstytucji 3 Maja 1/3, 96-100 Skierniewice, Poland; 2 Department of Horticultural Crop Breeding, The National Institute of Horticultural Research, Konstytucji 3 Maja 1/3, 96-100 Skierniewice, Poland; mariusz.lewandowski@inhort.pl

**Keywords:** red-fleshed apple clones, red-fleshed apple cultivar, freeze-drying, color features, computer vision, image parameters, sensory attributes

## Abstract

The target of breeding red-fleshed apples is to increase their potential health benefits related to red flesh coloration and consumer acceptance. The objective of this study was to determine the usefulness of four clones (90, 120, 156, and 158) of red-fleshed apples for freeze-drying compared to the cultivar ‘Trinity’. Red-fleshed apples were dried in the form of slices using a laboratory freeze-dryer. The changes in color features and image texture parameters after drying and the sensory quality of freeze-dried samples were assessed. Trends of increase in the value of the L* parameter and decrease in the a* and b* parameters after freeze-drying were observed. Furthermore, freeze-drying caused statistically significant changes in analyzed image textures named XHMean, RHMean, SHMean, VHMean, LHMean, and UHMean. Machine learning models developed based on the color parameters L*, a*, and b* distinguished raw and freeze-dried red-fleshed apples with an average accuracy of 84% for clone 90 up to 99.0% for clone 156, and models based on twenty selected image textures exhibited an accuracy of 98.5% for clone 156 to 100% for clones 90 and 158 and the cultivar ‘Trinity’. The very attractive external appearance, medium-intense fruity smell, crunchiness, and intense fruity taste of all the apple slices were revealed. The innovative aspect of this study included the comparison of the drying behavior and sensory quality of the new clones and a standard cultivar of red-fleshed apples. Moreover, innovative methods and results were used to determine the effect of freeze-drying on red-fleshed apple quality, considering novel models involving thousands of image textures and machine learning algorithms.

## 1. Introduction

Red-fleshed apples are known for their attractive color and high contents of health-promoting compounds, including polyphenols [1]. Red-fleshed apples may be used for consumption and nutraceutical purposes [2]. Consuming red-fleshed apples can inhibit the proliferation of cancer, reduce the risk of cardiovascular disease and inflammation, and influence immune function [3,4,5]. 

Organic acids are present in both the flesh and the peel, and malic acid is the main organic acid in red-fleshed apples. The high content of organic acids can cause them to be acidic and thus reduce consumer acceptance. Therefore, their poor taste can be improved by applying crossbreeding with good-flavored apples with white flesh to obtain new commercially viable cultivars. The breeding target is to increase the potential health benefits and consumer acceptance of red-fleshed apples. Furthermore, red-fleshed apples can be a biofortified crop [6]. Breeding programs focus on the development of cultivars of red-fleshed apples considering the association of red flesh coloration with high antioxidant activity and health-promoting effects by consumers. The successful positioning of new red-fleshed apple cultivars in a new market segment requires knowledge of consumer preferences to develop marketing strategies. In the apple market, consumer preferences are mainly related to fruit appearance, including shape, skin color, flesh color, and esthetic appeal, as well as taste, including, mouthfeel attributes, texture, and flavor [7]. Nowadays, marker-assisted breeding can accelerate the development of new cultivars of red-fleshed apples with outstanding parameters to ensure high-quality fruit accepted by consumers and appropriate production volumes. It can result in an increase in the consumption of red-fleshed apples, which is important for a healthy diet [8].

Drying is a promising procedure in apple processing and can allow for the production of healthy snacks with the retention of bioactive compounds and nutrients [9]. Additionally, drying is useful to decrease the weight and volume of fruit and thus reduce storage, packaging, and transportation costs [10]. Drying can be used to preserve red-fleshed apples and extend their shelf life. Drying reduces the water content to a level that inhibits the growth of microorganisms. However, it can cause changes related to the loss of pro-health compounds. Freeze-drying at a low temperature and reduced pressure can be the solution. This minimizes the loss of thermolabile compounds and results in only slight changes in the dried products [11]. Freeze-drying can be used to prepare functional and ready-to-eat foods with a minimum loss of aroma, flavor, and bioactive compounds and with excellent preservation results [12]. Assessment of the quality of dried final products is important to determine the impact of drying on changes in sample parameters. The morphological changes in the product, visual perception, and appearance to consumers can be assessed using machine vision systems. This can be of industrial relevance for processed apples, whose consumption as dried chips or snacks or as intermediate products is increasing [13]. Therefore, computer vision systems can be a perfect solution for non-destructive, automatic, cheap, sensitive, precise, and rapid monitoring of the quality of dried products [14]. Furthermore, machine learning models may be used in assessing post-harvest fruit quality for the classification; food grading and sorting; freshness assessment; determination of chemical and nutritional characteristics; detection of pathological disorders, damage, defects, and food contamination; as well as real-time monitoring of food in the supply chain and post-harvest loss mitigation [15,16]. 

This study was performed to evaluate changes in color parameters and image texture features, which are very important for consumer acceptance of dried apples, and to determine the sensory quality of freeze-dried red-fleshed apple clones and a cultivar. The quality assessment of apple samples combined external quality parameters, such as color and image textures, which indicated changes in the structure of apple flesh, measured using non-destructive and objective techniques, with sensory attributes assessed using a destructive and subjective approach. The innovative aspect of this study was related to the comparison of the drying behavior and sensory quality of new clones and a standard cultivar of red-fleshed apples. A novel approach involving thousands of image texture parameters and machine learning algorithms was applied to determine the effect of freeze-drying on red-fleshed apple quality.

## 2. Materials and Methods

### 2.1. Raw-Material and Freeze-Dried Red-Fleshed Apple Samples

The research material consisted of red-fleshed apples representing five genotypes, including the cultivar ‘Trinity’ and four clones numbered 90, 120, 156, and 158:

1. ‘Trinity’—standard cultivar;
2. Clone No. 90—the origin is not fully known, but ‘Trinity’ was used as the paternal (pollen) in the cross combination;
3. Clone No. 120—the origin is not fully known, but ‘Trinity’ was used as the paternal (pollen) in the cross combination;
4. Clone No. 156—origin (‘Ligol Red’ × ‘Trinity’);
5. Clone No. 158—origin (‘Rosana’ × ‘Trinity’).

Trees of these genotypes grow generally weakly or medium-strongly, forming a slightly conical crown that is medium-dense. They produce fruit that is small to medium-sized, spherical–conical, with a very attractive and uniform shape. The skin of the fruit is mostly covered with a dark red blush. The flesh is light red to dark red, juicy, with a slightly sour to sour taste. The fruits of these genotypes reach harvest maturity in the first half of September. The fruits store very well in both regular cold stores and cold stores with a controlled atmosphere (CA). These genotypes are not very susceptible to apple scab (*Venturia inaequalis*) and powdery mildew (*Podosphaera leucotricha*) and are winter-hardy in the weather conditions of central Poland.

Red-fleshed apple samples were subjected to freeze-drying using a laboratory dryer (LABCONCO, Kansas City, MO, USA). Apples were dried as slices with peel. Slices were characterized by a thickness equal to 4 mm. Before drying, sliced samples were frozen for 24 h in a freezer (Whirlpool) at a temperature of −28 °C. Frozen slices were spread on trays in one layer and put into a dryer. The initial temperature in the dryer was 20 °C. After the apple samples were placed in the dryer, the condenser temperature reached −55 °C. The process was carried out for 48 h. The pressure at the final stage of the process reached 6 kPa.

Fifty raw sliced apple samples and fifty freeze-dried samples of each cone and cultivar were prepared for color measurements and image acquisition. Additionally, freeze-dried apple slices were used to determine sensory quality attributes. 

### 2.2. Color Parameters

The measurements of the L* (lightness, from 0 (dark) to 100 (light)), a* (red (+)–green (−)), and b* (yellow (+)–blue (−)) color parameters [17] of the flesh of raw and freeze-dried apples were performed using the calibrated portable spectrophotometer Konica Minolta CM-2600d (Konica Minolta, Inc., Chiyoda-ku, Tokyo, Japan). Two measurements for each of the fifty apple slices of clones 90, 120, 156, and 158 and the cultivar ‘Trinity’ were taken using the CIE (Comission Internationale de l’Eclairage) standard illuminant D65. In total, the measurements were performed in 100 repetitions for each of the raw and freeze-dried samples. 

### 2.3. Image Processing 

**Figure 1:**
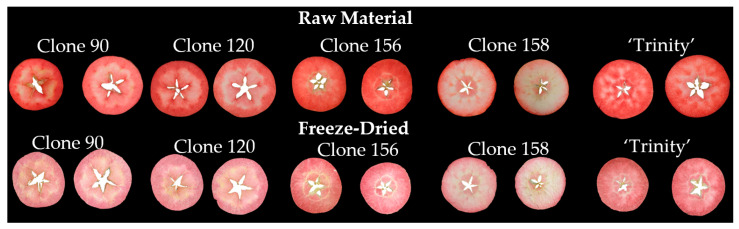
Exemplary images of red-fleshed apples before and after freeze-drying.

**Figure 2:**
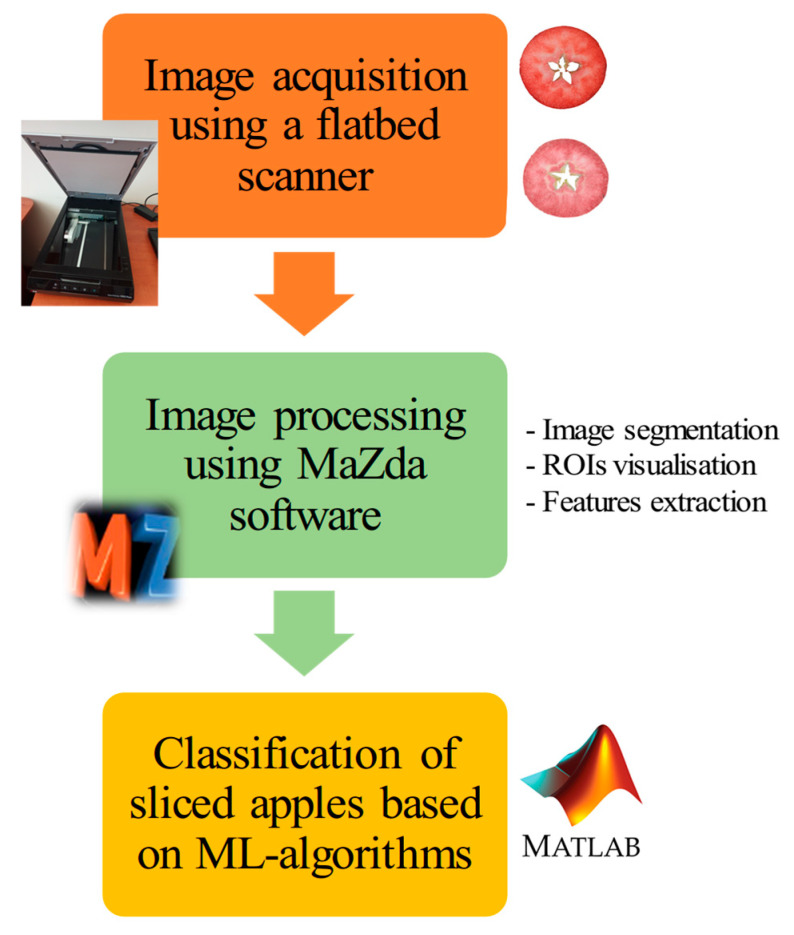
The flowchart of the research on the application of image texture parameters for distinguishing raw and freeze-dried red-fleshed apples.

Raw and freeze-dried samples of red-fleshed apples belonging to clones 90, 120, 156, and 158 and the cultivar ‘Trinity’ were imaged on a black background using an Epson Perfection flatbed scanner (Epson, Suwa, Nagano, Japan). Each of the 50 slices for each sample was imaged from both sides for a total of 100 repetitions. The images were saved in a TIFF format. The images of the slices of raw and freeze-dried red-fleshed apples are presented in Figure 1. After freeze-drying, there was a change in the color and a slight reduction in the diameter of the slices (shrinkage). However, there was no change in shape in terms of flatness and roundness. The images were processed using the MaZda 4.7 software (Łódź University of Technology, Institute of Electronics, Łódź, Poland) [18,19,20]. The images were segmented using brightness thresholding. Lighter red-fleshed apple slices were separated from the black background and treated as regions of interest. In the case of each color channel of the images, such as *X*, *Y*, *Z*, *R*, *G*, *B*, *S*, *V*, *L*, *a*, *b*, and *U*, 181 image texture parameters were calculated based on the co-occurrence matrix (132 textures), run-length matrix (20 textures), histogram (9 textures), Haar wavelet transform (10 textures), autoregressive model (5 textures), and gradient map (5 textures) [17]. Thus, for each apple slice image, in total, 2172 texture features were determined. The textures extracted from the red-fleshed apple images were treated as a function of the spatial variation in the pixel brightness intensity. Texture carries information about sample structure. The quantitative analysis of image textures provides insights into sample quality. Images exhibit repeated subpatterns of the distribution and dispersion of pixel brightness, which represent the color, roughness, brightness, smoothness, directivity, size, and granulation of textures. For classification, only selected textures are characterized by significant information. Therefore, only the most desirable textures are chosen before further analysis [18,19,20,21,22]. The flowchart of distinguishing raw and freeze-dried red-fleshed apple samples based on image texture parameters is presented in Figure 2.

### 2.4. Evaluation of the Effect of Freeze-Drying on the Appearance of Red-Fleshed Apple Flesh Using Machine Learning Models

The effects of freeze-drying of red-fleshed apple slices on color parameters and image textures were determined using machine learning algorithms. Models were developed separately for each apple clone and the cultivar ‘Trinity’, with the results obtained for raw and dried samples included in each model. Machine learning models were developed based on three color parameters, L*, a*, and b*, and then texture features were selected from a set of 2172 attributes. The image texture selection was performed using the ReliefF algorithm, and 20 features were selected for each model. Models were built using MATLAB R2024a (MathWorks, Inc., Natick, MA, USA). A mode of 10-fold cross-validation and Neural Network, SVM (Support Vector Machine), KNN (K-Nearest Neighbors), Naive Bayes, Discriminant Analysis, Tree, and Ensemble algorithms were used. The models with the highest classification accuracies were selected. In the case of the models developed based on color parameters, all color parameters, L*, a*, and b*, were included in each model, and the following model hyperparameters were used for distinguishing raw and freeze-dried samples of the individual clones and the cultivar:

- Clone 90—Preset: Quadratic SVM; Kernel scale: Automatic; Kernel function: Quadratic; Multiclass method: One-vs-One; Box constraint level: 1; Standardized data: Yes;
- Clone 120—Preset: Weighted KNN; Number of neighbors: 10; Distance weight: Squared inverse; Distance metric: Euclidean; Standardized data: Yes;
- Clone 156—Preset: Narrow Neural Network; Layer size: 10; Number of fully connected layers: 1; Iteration limit: 1000; Regularization strength (Lambda): 0; Activation: ReLU; Standardized data: Yes;
- Clone 158—Preset: Medium Gaussian SVM; Kernel scale: 1.7; Kernel function: Gaussian; Multiclass method: One-vs-One; Box constraint level: 1; Standardized data: Yes;
- Cultivar ‘Trinity’—Preset: Medium Gaussian SVM; Kernel scale: 1.7; Kernel function: Gaussian; Multiclass method: One-vs-One; Box constraint level: 1; Standardized data: Yes.

In the case of the models that distinguished raw and freeze-dried apple samples using selected image features, the following hyperparameters were applied to build the models:

- Clone 90—Preset: Fine Gaussian SVM; Kernel scale: 1.1; Kernel function: Gaussian; Multiclass method: One-vs-One; Box constraint level: 1; Standardized data: Yes;
- Clone 120—Preset: Linear Discriminant; Covariance structure: Full;
- Clone 156—Preset: Fine Tree; Maximum number of splits: 100; Split criterion: Gini’s diversity index; Surrogate decision splits: Off;
- Clone 158—Preset: Gaussian Naive Bayes; Distribution name for numeric predictors: Gaussian; Distribution name for categorical predictors: Not applicable;
- Cultivar ‘Trinity’—Preset: Kernel Naive Bayes; Distribution name for numeric predictors: Kernel; Distribution name for categorical predictors: Not applicable; Kernel type: Gaussian; Support: Unbounded.

For the models, the overall accuracies, prediction speeds, training times, and confusion matrices were determined. 

### 2.5. Sensory Analysis

Sensory attributes were assessed for the freeze-dried red-fleshed apples. The determined parameters are presented in Table 1. Quantitative Descriptive Analysis (QDA) was carried out in a laboratory room with four booths containing computers and white light sources according to PN-EN ISO 8589:2010/A1:2014-07 [23] and PN-EN ISO 13299:2016-05 [24]. Freeze-dried red-fleshed apple samples were assessed by 10 panelists qualified in accordance with PN-EN ISO 8586:2014-03 [25]. Five covered plastic containers holding five slices of red-fleshed apples of clones 90, 120, 156, and 158 and the cultivar ‘Trinity’ were presented to each person at the same time to allow the comparison of apple sensory quality. Samples were marked with a three-digit code. Non-carbonated mineral water was used as a taste neutralizer.

### 2.6. Mean Comparison

The means of the color parameters and selected image textures of raw and freeze-dried red-fleshed apples were compared to determine the effect of drying on the external quality of the samples. Additionally, the sensory attributes of the clones and the cultivar of freeze-dried apples were compared. Analysis was performed using STATISTICA 13.1 (Dell Inc., Tulsa, OK, USA, StatSoft Polska, Kraków, Poland). The normality of the variable distribution and homogeneity of variance were checked. Tukey’s test was used for the mean comparison. A significance level of *p* < 0.05 was applied.

## 3. Results

### 3.1. Color Parameters of Raw and Freeze-Dried Red-Fleshed Apple Slices

The comparison of the color parameters L*, a*, and b* between the raw and freeze-dried red-fleshed apple samples is presented in Table 2. The color of the raw apples depended on their pedigree, which also influenced the color of the samples after drying. In the case of each clone, 90, 120, 156, and 158, and the cultivar ‘Trinity’, statistically significant changes in lightness (L*) after drying were observed. Freeze-dried samples were characterized by higher values of the L* parameter. This meant that the dried samples were lighter. The values of a* were positive (+) for all raw and dried apples, which indicated the red color. For all analyzed apple clones, a statistically significant decrease in the parameter a* was found after drying. The positive (+) values of b* were related to the yellowness of all the apple samples. Freeze-drying statistically significantly decreased the b* values for clones 120 and 156 and the cultivar ‘Trinity’.

### 3.2. Image Texture Features of Raw and Freeze-Dried Apple Samples

From a set of extracted image texture parameters, the differences in the texture HMean (histogram mean) for selected color channels *(X*, *R*, *S*, *V*, *L*, and *U*) between raw and dried red-fleshed apples are shown in Table 3. It was found that freeze-drying caused statistically significant changes in all analyzed image textures, XHMean, RHMean, SHMean, VHMean, LHMean, and UHMean. In the case of texture features of XHMean, RHMean, LHMean, and UHMean, drying resulted in an increase in the values of these parameters, whereas for SHMean and VHMean, freeze-dried samples were characterized by lower values than the raw material.

### 3.3. Machine Learning Models for Distinguishing Raw and Freeze-Dried Red-Fleshed Apple Slices Based on Color and Image Texture Parameters

**Figure 3:**
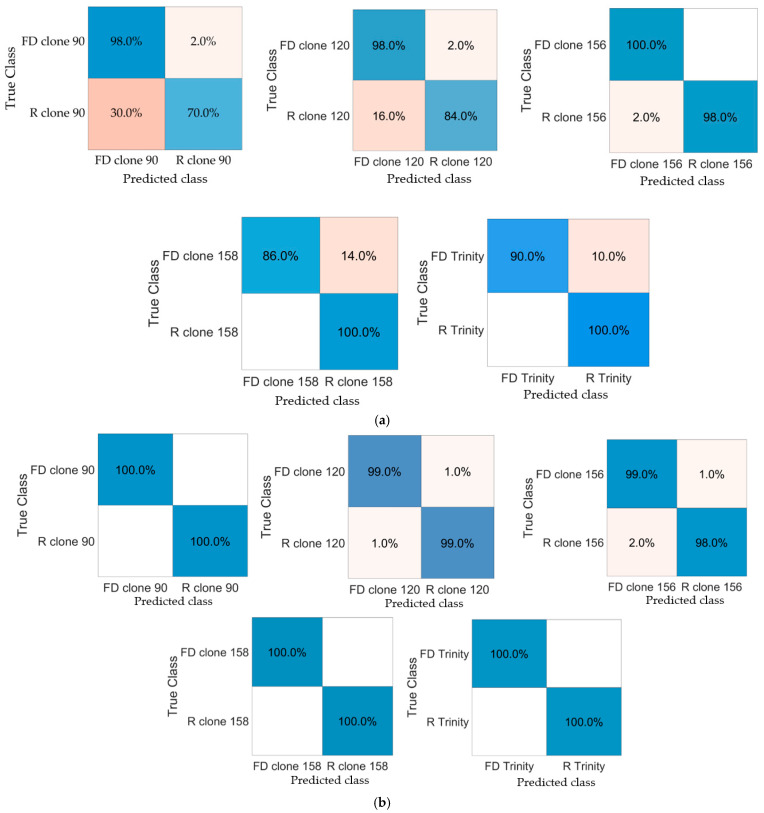
The classification of raw and freeze-dried red-fleshed apples using machine learning models built based on color parameters (**a**) and image texture features (**b**). FD—freeze-dried; R—raw material.

Additionally, for the mean comparison of parameters presented in Table 2 and Table 3, machine learning models were developed to distinguish raw and freeze-dried red-fleshed apples based on color (Figure 3a) and selected image textures (Figure 3b). In the case of color, all parameters, L*, a*, and b*, were included in each model, while for image textures, for each model, twenty attributes were selected from a set of 2172 textures. The exemplary textures with the highest discriminative power for clone 90 were as follows: GHPerc01, LHKurtosis, aHMean, aATeta1, bS5SH1SumAverg, YHKurtosis, ZS5SV1SumEntrp, ZS5SZ3AngScMom, UHPerc90, VHSkewness; for clone 120: RHSkewness, aHPerc90, aATeta3, bS4RVRLNonUni, bATeta4, ZS5SN1Entropy, ZS5SV5AngScMom, UHPerc50, US5SH5AngScMom, SHVariance; for clone 156: RS5SV5Correlat, GATeta2, BS5SN1SumOfSqs, LSGNonZeros, bATeta2, YHVariance, ZHDomn10, UATeta1, VS5SV1DifVarnc, SHPerc50; for clone 158: RSGArea, RS5SZ5AngScMom, BHVariance, BS5SN3AngScMom, aATeta3, bATeta2, ZS5SV1SumEntrp, ZS5SH5SumAverg, UHPerc99, SHVariance; and for the cultivar ‘Trinity’: RS5SV1SumOfSqs, RS5SZ5AngScMom, GHPerc01, LHMean, aATeta4, bHPerc90, bHDomn01, bATeta2, XS5SZ5SumAverg, ZS5SN1SumEntrp. Color characteristics allowed for the classification of the raw and dried apples with high correctness (Figure 3a). The average accuracy ranged from 84% for clone 90 to 99.0% for clone 156. In the case of clone 90, the most successful model was built using Quadratic SVM, the prediction speed was equal to 1300 observations/second (obs/s), and the training time was 8.96 s. For clone 156, the model built using a Narrow Neural Network was characterized by a prediction speed of 930 obs/s and a training time of 8.99 s. The machine learning model that distinguished raw and freeze-dried samples of red-fleshed apples belonging to clone 120 provided an accuracy of 91% (Weighted KNN) with a speed of 220 obs/s and a training time equal to 4.00 s. Raw and dried samples of clone 158 were classified with an accuracy of 93.0% (Medium Gaussian SVM), a prediction speed of 1400 obs/s, and a training time of 3.46 s. The classification model developed for raw and freeze-dried ‘Trinity’ apples was characterized by an accuracy equal to 95.0% (Medium Gaussian SVM), a prediction speed of 1200 obs/s, and a training time of 6.92 s. 

Models built based on selected textures classified raw and freeze-dried red-fleshed apple slices with an average accuracy reaching 100% for clone 90 using the Fine Gaussian SVM algorithm, clone 158 using Gaussian Naive Bayes, and ‘Trinity’ using Kernel Naive Bayes (Figure 3b). In the case of clone 90, a developed model was characterized by a prediction speed of 250 obs/s and a training time of 688.03 s; for clone 158, a prediction speed of 180 obs/s and a training time of 427.94 s; and for ‘Trinity’, a prediction speed of 170 obs/s and a training time of 457.10 s. Raw and dried samples of clone 120 were distinguished at a rate of 99.0% (Linear Discriminant), with a prediction speed of 260 obs/s and a training time of 96.71 s, and samples of clone 156 were distinguished at a rate of 98.5% (Fine Tree), with a prediction speed of 180 obs/s and a training time of 11.06 s.

### 3.4. Sensory Quality of Red-Fleshed Apples

**Figure 4:**
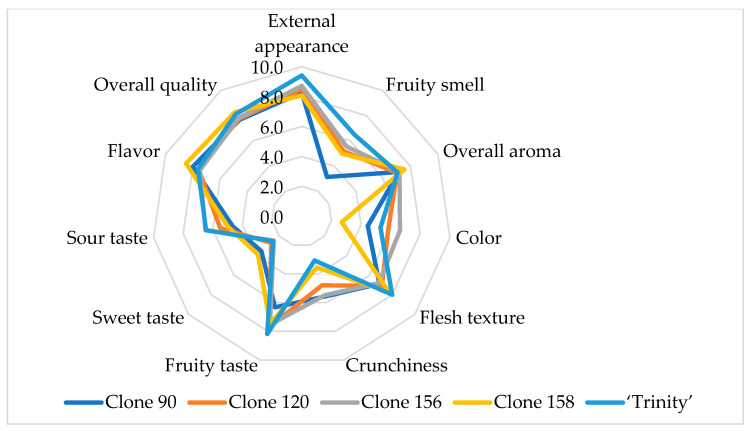
Radar chart of the sensory attributes of freeze-dried red-fleshed apples belonging to different clones and the cultivar ‘Trinity’.

The sensory attributes related to the appearance, aroma, taste, and flavor of red-fleshed apple slices belonging to four clones and one cultivar are presented in Table 4 and Figure 4. All apple samples were characterized by a very attractive external appearance, from 8.1 for clone 158 to 9.4 for ‘Trinity’ on a scale from 0 to 10, a medium-intense fruity smell from 4.6 for clone 90 to 6.5 for ‘Trinity’, an almost imperceptible off-odor, and a pleasant overall aroma with a value of about 7.0. The differences in these parameters between samples were not statistically significant, whereas freeze-dried apple slices were statistically significantly different in terms of the color of the flesh. One homogenous group with the darkest flesh included clone 156 (6.6), clone 120 (5.8), and ‘Trinity’ (5.3). The lightest color was found for clone 158 (2.7). The remaining sensory attributes were not statistically significantly different between samples. The flesh texture of all samples was hard, in the range of 6.8 for clone 156 to 8.0 for ‘Trinity’. Apple slices were crunchy with a loud sound. Samples were characterized by an intense fruity taste from 7.3 for clone 90 to 8.2 for ‘Trinity’, a not intense sweet taste from 2.5 for clone 156 and ‘Trinity’ to 3.9 for clone 158, and a medium-intense sour taste from 4.7 for clones 90 and 158 to 6.5 for clone 156 and ‘Trinity’. Bitter taste and off-flavor were not found. Freeze-dried apple slices were characterized by a rich, aromatic, and palatable flavor, reaching 8.5 for clone 158 and a very good overall quality of up to 8.3 for clone 158.

## 4. Discussion

In the present study, the effect of freeze-drying on the color and image texture parameters of the cultivar ‘Trinity’ and all new clones of red-fleshed apples was revealed. It was indicated that the visual properties and structure of the flesh of apples were changed after drying. Kidoń and Grabowska [11] compared the effect of freeze-drying, convective drying, and vacuum-microwave combined with convective drying on the properties of red-fleshed apple cubes, such as color and sensory attributes. The freeze-dried apple samples were characterized by the highest L* and a* color parameters and the most desirable sensory qualities related to color and odor. Moreover, similarly to our study, a higher value of the L* parameter was found by Kidoń and Grabowska [11] for freeze-dried apples than for fresh ones, which confirms the influence of freeze-drying on the brightening of the products. Obtaining the highest-quality products in this way may be due to the fact that freeze-drying is a non-thermal processing technology [12]. Unfavorable color changes in red-fleshed apples may increase with increasing drying temperature [26]. Freeze-drying can allow for obtaining final products of very high quality compared with other drying methods. However, freeze-drying has some limitations. It is characterized by a long time and high energy consumption. Therefore, the combination of freeze-drying with other drying methods can be used to reduce energy consumption and shorten the drying rate while maintaining the high quality of products [27,28]. 

The obtained results may be of great practical importance. The main objective of the research is to produce dessert cultivars of red-fleshed apples with properties that are highly acceptable to potential consumers. The breeding program is aiming at producing red-fleshed dessert apples with a more sweet and less sour taste and attractive red flesh color related to the high antioxidant activity. The practical goal of the present study was to develop an objective and non-destructive procedure for the assessment of the changes in red-fleshed apple quality after freeze-drying. The image analysis combined with machine learning proved to be useful for this purpose. Therefore, the developed procedure may have practical applications in red-fleshed apple breeding programs and processing. The present study revealed the very good sensory quality of freeze-dried red-fleshed clones, which was confirmed by their attractive external appearance and rich, aromatic, and palatable flavor. The sensory attributes for some clones were more desirable than for the standard cultivar ‘Trinity’. These results are very promising with respect to continuing the breeding program to obtain new red-fleshed apple cultivars with the best appearance and taste for consumers. The studies involving sensory analysis were carried out only with the participation of trained experts, who were specially selected for their taste and smell sensitivity for this type of product. This gave reliable results and a general view of the effectiveness of clone breeding aimed at obtaining dessert cultivars of red-fleshed apples. In addition to appearance and flavor, the nutritional value of new clones and cultivars is also significant. Therefore, nutritional analysis, evaluation of chemical composition, and antioxidant analysis will be performed for the same samples. Considering color, structure, and sensory attributes, it will be possible to select the most promising clones, which will be presented to consumers. Therefore, research will be continued in the next growing seasons to perform consumer acceptance tests too. 

Freeze-dried apple chips, due to the low temperature of drying, have a highly porous and fragile structure, relatively low hardness, and low bulk density. These observations occur because, during the process, intercellular spaces and plant cells are intact due to the sublimation of ice crystals. Large pores may be caused by the formation of large ice crystals. Furthermore, chemical reactions, including pectin solubilization, during freeze-drying are slow. Additionally, structural deformations can be visible in freeze-dried apple slices compared with raw material as a result of the loss of the organized structure of the tissue due to texture breakages, collapsed cells, and the formation of large intercellular spaces [29,30,31]. In our study, image texture parameters were used to determine the changes in the red-fleshed apple structures caused by freeze-drying. The present study is an extension of our previous research on red-fleshed apples and has been developed in other research directions. The present article evaluated the changes in color features and image texture parameters after freeze-drying and the sensory quality of freeze-dried samples. Thus, the features of apples were compared between raw-material and freeze-dried samples. This reflected the changes in the quality of red-fleshed apples caused by drying. Additionally, the sensory attributes of the freeze-dried samples were examined as part of a preliminary assessment of the human acceptability of the products. This will enable the selection of clones to continue breeding programs aimed at developing dessert varieties with desired properties for processing, allowing the production of high-quality ready-to-eat products. A previous article by Ropelewska et al. [32] about red-fleshed apples focused only on the discrimination (distinguishment) of ‘Alex Red’, ‘Trinity’, ‘314′, and ‘602′ red-fleshed apple samples based on selected textures of images or features from a set combining color parameters and image textures.

Research on post-harvest treatment of red-fleshed apples is very important. The post-harvest management of red-fleshed apples is challenging compared to that of conventional white-fleshed apples. Red-fleshed apples require different post-harvest and storage treatments to white-fleshed apples because the fruit quality easily degrades [33]. Red-fleshed apples with a dark flesh color may exhibit more flesh browning during storage than apples with a lighter red flesh color [34]. The risk of internal browning of red-fleshed apples with high anthocyanin contents and internal ethylene concentrations may be high. Ethylene concentration determines apple shelf life and storability. Due to their high ethylene content, red-fleshed apples lose their firmness and crispness during storage, which reduces their quality. Appropriate specialized storage can slightly extend their shelf life [35]. Additionally, to increase the shelf life and stability and minimize changes in the nutritional value of red-fleshed apples, post-harvest treatment in the production of freeze-dried snacks is also a good solution [36]. 

Non-destructive quality assessment of apples is important. Image analysis may be considered a superior approach to product quality evaluation compared to conventional color measurements and organoleptic analysis of visual attributes. In the case of color, which is a main factor in the evaluation of food quality, panelists’ perceptions of it during sensory analysis are subjective. This was a limitation of this assessment. Spectrophotometric measurements do not provide information about color distribution. Image processing enables objective evaluation of color heterogeneity and discrimination of samples. Detailed information provided about color distribution is relevant for the analysis of the overall quality of products [37]. Image analysis and machine learning were also used in other studies on apples. For example, Zhang et al. [38] used color–texture features in apple images to segment fruit in orchards by machine learning algorithms, obtaining an accuracy of up to 0.94. Texture and color features computed from images were also used to classify ten apple cultivars using machine learning models with an average accuracy reaching more than 98% [39]. In addition to traditional machine learning, deep learning is also useful for distinguishing apple cultivars [40]. Furthermore, image processing combined with machine learning was applied to predict the development of apple skin color with an accuracy of more than 96% [41]. Generally, in food processing, the application of machine learning and deep learning may improve production efficiency and outcomes [42]. The importance of machine learning in supply chains is increasing. This may affect the resilience and sustainability of entire supply chains [43].

## 5. Conclusions

The performed study revealed the effect of freeze-drying on the quality of new clones of red-fleshed apples and the cultivar ‘Trinity’ in terms of color parameters and image textures. Freeze-drying caused changes in the visual properties and flesh structure of apple slices. This was reflected in the differences in the L*, a*, and b* color parameters and textures in images in various color channels between the raw and dried samples. The application of machine learning models based on color and image texture parameters allowed us to demonstrate the great influence of freeze-drying on red-fleshed apple slices in a non-destructive and objective manner. Additionally, the sensory attributes related to the external appearance, smell, aroma, color, taste, and flavor of dried apples were assessed. Subjective assessment of sensory attributes revealed a palatable flavor and good overall quality for all the freeze-dried red-fleshed apple samples. Research can be continued by combining freeze-drying with other types of drying, as well as by determining the effect of drying on the physicochemical properties of red-fleshed apples belonging to new clones and cultivars.

## Figures and Tables

**Table 1:** Sensory attributes of freeze-dried red-fleshed apples.

Attribute	Boundary Values	
	0—Minimum	10—Maximum
External appearance	Non attractive	Very attractive
Fruity smell	Imperceptible	Very intense
Off-odor	Imperceptible	Very intense
Overall aroma	Unpleasant, irritating	Very pleasant
Color	Bright	Dark
Flesh texture	Very hard	Soft
Crunchiness	No sound	Loud sound
Fruity taste	Imperceptible	Very intense
Sweet taste	Imperceptible	Very intense
Sour taste	Imperceptible	Very intense
Bitter taste	Imperceptible	Very intense
Off-flavor	Imperceptible	Very intense
Flavor	Empty, uncharacteristic	Rich, aromatic, palatable
Overall quality	Poor quality	Very good quality

**Table 2:** Color parameters of raw and freeze-dried red-fleshed apple samples.

Samples of Red-Fleshed Apple Slices	L*	a*	b*
Raw clone 90	56.94 (6.42) ^a^	25.94 (8.81) ^a^	16.36 (4.73) ^a^
Freeze-dried clone 90	62.47 (3.69) ^b^	20.23 (3.94) ^b^	15.97 (5.77) ^a^
Raw clone 120	55.04 (6.70) ^a^	30.06 (6.77) ^a^	14.79 (4.16) ^a^
Freeze-dried clone 120	61.67 (5.29) ^b^	20.69 (3.40) ^b^	11.84 (4.34) ^b^
Raw clone 156	49.61 (6.95) ^a^	35.33 (6.23) ^a^	16.85 (2.31) ^a^
Freeze-dried clone 156	59.23 (7.08) ^b^	24.02 (7.70) ^b^	10.99 (5.05) ^b^
Raw clone 158	63.80 (7.91) ^a^	17.53 (8.27) ^a^	10.99 (3.73) ^a^
Freeze-dried clone 158	73.11 (5.52) ^b^	14.47 (4.85) ^b^	11.79 (5.01) ^a^
Raw ‘Trinity’	51.96 (8.06) ^a^	33.96 (9.78) ^a^	13.47 (4.25) ^a^
Freeze-dried ‘Trinity’	59.87 (7.28) ^b^	31.69 (6.81) ^a^	3.35 (3.32) ^b^

^a,b^—The same letters in the columns for raw and freeze-dried samples of each clone and cultivar mean no statistical differences; values in parentheses are standard deviations.

**Table 3:** Selected image texture features of raw and freeze-dried red-fleshed apples.

Sample of Red-Fleshed Apple Slices	XHMean	RHMean	SHMean	VHMean	LHMean	UHMean
Raw clone 90	95.66 (15.23) ^a^	210.45 (20.75) ^a^	100.00 (18.64) ^a^	174.77 (12.01) ^a^	166.10 (10.17) ^a^	104.91 (5.27) ^a^
Freeze-dried clone 90	119.42 (19.11) ^b^	216.08 (13.06) ^b^	65.33 (5.79) ^b^	156.87 (2.33) ^b^	189.36 (10.77) ^b^	110.19 (3.56) ^b^
Raw clone 120	97.30 (19.33) ^a^	204.37 (16.17) ^a^	98.32 (14.70) ^a^	174.54 (9.29) ^a^	167.12 (12.69) ^a^	105.26 (2.48) ^a^
Freeze-dried clone 120	104.28 (19.30) ^b^	211.52 (21.26) ^b^	63.53 (7.09) ^b^	157.29 (3.63) ^b^	179.14 (12.87) ^b^	112.66 (2.95) ^b^
Raw clone 156	85.17 (17.03) ^a^	208.20 (20.16) ^a^	117.75 (18.87) ^a^	184.15 (11.57) ^a^	155.45 (13.21) ^a^	101.44 (2.72) ^a^
Freeze-dried clone 156	108.42 (18.12) ^b^	212.36 (12.71) ^b^	74.08 (9.55) ^b^	163.12 (5.47) ^b^	179.24 (12.31) ^b^	111.71 (2.60) ^b^
Raw clone 158	96.25 (25.71) ^a^	191.52 (32.92) ^a^	62.77 (17.05) ^a^	153.89 (10.73) ^a^	173.23 (21.26) ^a^	108.98 (6.09) ^a^
Freeze-dried clone 158	108.76 (13.76) ^b^	202.56 (10.20) ^b^	53.57 (6.91) ^b^	151.05 (4.08) ^b^	185.30 (9.07) ^b^	112.68 (2.19) ^b^
Raw ‘Trinity’	88.85 (18.63) ^a^	207.77 (25.59) ^a^	110.57 (25.25) ^a^	180.70 (16.00) ^a^	158.81 (12.08) ^a^	104.27 (4.77) ^a^
Freeze-dried ‘Trinity’	104.66 (19.44) ^b^	214.30 (12.24) ^b^	86.00 (10.25) ^b^	170.36 (5.35) ^b^	172.91 (13.84) ^b^	112.32 (2.27) ^b^

^a,b^—The same letters in the columns for raw and freeze-dried samples of each clone and cultivar mean no statistical differences; values in parentheses are standard deviations.

**Table 4:** Sensory attributes of freeze-dried red-fleshed apples.

Freeze-Dried Apple	External Appearance	Fruity Smell	Off-Odor	Overall Aroma	Color	Flesh Texture	Crunchiness	Fruity Taste	Sweet Taste	Sour Taste	Bitter Taste	Off-Flavor	Flavor	Overall Quality
Clone 90	8.3 (1.0) ^a^	4.6 (2.1) ^a^	0.9 (0.6) ^a^	7.1 (1.7) ^a^	4.5 (1.7) ^ab^	6.9 (1.4) ^a^	7.0 (2.3) ^a^	7.3 (2.7) ^a^	3.6 (2.1) ^a^	4.7 (1.9) ^a^	0.0 (0.0) ^a^	0.0 (0.0) ^a^	8.0 (0.7) ^a^	7.7 (0.6) ^a^
Clone 120	8.5 (1.4) ^a^	5.2 (2.4) ^a^	0.2 (0.5) ^a^	7.1 (1.5) ^a^	5.8 (2.1) ^b^	7.1 (1.4) ^a^	6.8 (2.5) ^a^	7.7 (1.0) ^a^	2.7 (2.2) ^a^	5.5 (2.2) ^a^	0.0 (0.0) ^a^	0.0 (0.0) ^a^	7.6 (1.1) ^a^	7.7 (0.9) ^a^
Clone 156	8.7 (0.6) ^a^	5.5 (2.3) ^a^	0.0 (0.0) ^a^	7.2 (1.5) ^a^	6.6 (1.6) ^b^	6.8 (1.5) ^a^	7.2 (2.0) ^a^	7.5 (1.2) ^a^	2.5 (2.3) ^a^	6.5 (1.7) ^a^	0.0 (0.0) ^a^	0.0 (0.0) ^a^	7.5 (1.4) ^a^	7.8 (1.4) ^a^
Clone 158	8.1 (1.1) ^a^	5.0 (2.5) ^a^	0.1 (0.1) ^a^	7.5 (1.3) ^a^	2.7 (1.7) ^a^	7.7 (1.7) ^a^	6.2 (2.6) ^a^	7.5 (1.4) ^a^	3.9 (2.2) ^a^	4.7 (2.4) ^a^	0.0 (0.0) ^a^	0.0 (0.0) ^a^	8.5 (1.1) ^a^	8.3 (1.0) ^a^
‘Trinity’	9.4 (0.5) ^a^	6.5 (1.0) ^a^	0.0 (0.0) ^a^	7.0 (3.2) ^a^	5.3 (3.9) ^b^	8.0 (1.3) ^a^	6.0 (2.8) ^a^	8.2 (1.5) ^a^	2.5 (2.4) ^a^	6.5 (1.8) ^a^	0.0 (0.0) ^a^	0.0 (0.0) ^a^	7.6 (1.4) ^a^	8.1 (0.8) ^a^

^a,b^—The same letters in the columns for clones and cultivar of freeze-dried apple samples mean no statistical differences; values in parentheses are standard deviations.

## Data Availability

The original contributions presented in the study are included in the article. Further inquiries can be directed to the corresponding author.
